# Secularization, Union Formation Practices, and Marital Stability: Evidence from Italy

**DOI:** 10.1007/s10680-012-9255-4

**Published:** 2012-02-15

**Authors:** Roberto Impicciatore, Francesco C. Billari

**Affiliations:** 1 Department of Economics, Business and Statistics, Università degli Studi di Milano, Via Conservatorio, 7, 20122 Milan, Italy; 2 Carlo F. Dondena Centre for Research on Social Dynamics, Università Bocconi, Via Guglielmo Röntgen 1, 20136 Milan, Italy; 3 Department of Policy Analysis and Public Management and IGIER, Università Bocconi, Via Guglielmo Röntgen 1, 20136 Milan, Italy

**Keywords:** Marital instability, Pre-marital cohabitation, Civil marriage, Religion, Multiprocess models, Selectivity, Instabilité conjugale, Cohabitation pré-maritale, Mariage civil, Religion, Modèles à équations simultanées, Sélection

## Abstract

Descriptive statistics indicate that civil marriages and marriages preceded by premarital cohabitation are more unstable, i.e., more frequently followed by divorce. However, the literature has shown that selectivity plays an important role in the relationship between premarital cohabitation and union dissolution. We do not have evidence to date regarding the selectivity in the effect of civil marriage. The Italian case appears particularly interesting given the recent diffusion of premarital cohabitation and civil marriage. Using micro-level data from a national-level representative survey conducted in 2003, we develop a multiprocess model that allows unobserved heterogeneity to be correlated across the three decisions (premarital cohabitation, civil marriage, and divorce). Our results show that selectivity is the main factor that explains the higher divorce rates among those who experience premarital cohabitation and a civil marriage. Net of selectivity, the causal effect on union dissolution disappears.

## Résumé

D’après des statistiques descriptives, les mariages civils et les mariages précédés d’une cohabitation pré-maritale sont plus instables, c’est-à-dire plus fréquemment rompus par un divorce. La littérature a cependant relevé que la sélection joue un rôle important dans la relation entre la cohabitation pré-maritale et la rupture d’union. Jusqu’à présent, un tel effet de sélection n’a pas été décrit pour le mariage civil. Le cas de l’Italie s’avère particulièrement intéressant du fait de la diffusion récente de la cohabitation pré-maritale et du mariage civil. A partir des données individuelles d’une enquête représentative au niveau national conduite en 2003, nous développons un modèle à équations simultanées permettant de tenir compte de l’hétérogénéité non observée corrélée aux trois comportements (cohabitation pré-maritale, mariage civil et divorce). Les résultats montrent que l’effet de sélection est le principal facteur explicatif de taux de divortialité plus élevés parmi ceux qui se sont mariés civilement après une période de cohabitation. En l’absence de sélection, l’effet causal de ces comportements sur la rupture d’union disparaît.

## Introduction

Changes in family and fertility behaviors in the western world have been strongly linked to secularization (defined, for simplicity, as an overall reduction in religious practice; for a critical analysis of the concept of secularization see, among others, Norris and Inglehart 2004; Gorski and Altınordu 2008), to the withdrawal from traditional religious beliefs, and to a decline in subjective religiosity among individuals (Lesthaeghe and Surkyn 1988; Van de Kaa 1987; Lesthaeghe and Neidert 2006). The literature, in which the idea of “Second Demographic Transition” plays a pivotal role, emphasizes the importance of the ideational shift from the influence of normative authorities to individual autonomy and the rejection of irreversible choices in shaping current trends in North America and Western Europe.

Less is known on the specific mechanisms through which declining religiosity, or more specifically secularization, affect family and fertility change. In what follows we aim to disentangle these mechanisms, focusing on a specific context, i.e., contemporary Italy, a particularly relevant setting for such a study. In Italy, the Roman Catholic Church is dominant and still has a strong influence, both on political decisions and on (some) actual individual behaviors. Premarital sex, cohabitation, and divorce are forbidden by the Church, whereas a high value is placed on marriage and family life. More generally, religion and religiousness still play an important role in Italy, as underlined by several studies. Starting from the three waves of the World Values Surveys between 1981 and 2001 which contain numerous questions on religiosity, Norris and Inglehart (2004) confirm the downward trend in religious participation in a large number of countries, except in Italy, Ireland, and the United States. Greeley (2002) analyzes four surveys carried out between 1980 and 1998, showing that in Italy religion has remained widespread and stable. However, a noticeable diffusion of “new” choices such as (premarital) cohabitation, civil marriage, extramarital childbearing, and divorce is taking place. Despite its slow secularization, Italy was a prime example of “lowest-low” fertility levels during the 1990s (Kohler et al. 2002).

In what follows, we focus on marital stability as a key life course outcome. How is this outcome influenced by secularization in a society like Italy? Before answering this question, how do we actually identify secularization? Usually, religiosity is measured through denomination (which in this case does not vary substantially), subjective statements (e.g., self-assessed religiosity), or specific behaviors such as frequency of prayer, attending services, and participation in religious social events. There are two limitations in measuring religiosity this way. First, the measure is intrinsically subjective and it may reflect not only actual religiosity but also what is socially acceptable in a specific context. Thus, in an area characterized by a highly normative participation in religious events, the effect on behaviors might be overestimated. Second, in a retrospective survey we have information on behaviors only related to the time at interview. Therefore, religious participation observed at the interview could be influenced by life-course choices, including marriage, cohabitation, and divorce (Thornton et al. 1992). These limitations can be overcome using panel data, but only if panel waves cover a long period of time, e.g., some decades, in order to evaluate the risk of disruption for a cohort of marriages. There are not many choices in the life of an individual that constitute an unequivocal manifestation of religiosity. Focusing on married people, one “external” expression of religiosity versus secularization is the binary choice between a civil (i.e., non-religious) and a religious marriage. Especially in Italy, where religious marriages are still predominant, a civil marriage clearly represents a secularized choice, displayed to the outside world.^1^ As a mere statistical indicator of behavior, civil marriage can unambiguously be detected using retrospective interviews. We can therefore expect couples opting for civil marriage to be potentially selected for a “higher-risk” marriage. This aspect is much less widely documented and analyzed than premarital cohabitation (see, e.g., Dittgen 1995). In general, civil marriage may have the same role as premarital cohabitation because it reflects the “new” attitudes and values that characterize a secularized society. Not only are these behaviors closely linked (cohabitors are also more likely to choose civil marriage) but they also potentially have long-term consequences since they do not imply religious commitment for the couple and their children. This is a sensitive issue for the Catholic Church agenda that very often promotes public discussion about the importance of religious marriage as the unique form of union.

The aim of this article is to study the relationship between premarital cohabitation (vs. a direct marriage), civil marriage (vs. a religious marriage), and the stability of the subsequent marriage. We see both premarital cohabitation and civil marriage as open manifestations of secularization at the individual (or, better, couple) level. The impact of premarital cohabitation on subsequent union instability has been investigated by several authors (see, among others, Lillard et al. 1995; Axinn and Thornton 1992; Berrington and Diamond 1999; Hoem and Hoem 1992; Teachman et al*.* 1991; Hall and Zhao 1995; Bracher et al. 1993; Bennet et al. 1988; De Maris and Rao 1992; Thomson and Colella 1992) whereas little, if any, attention has been paid to the effect of civil marriage. More specifically, we aim to separate two components, i.e., whether secularized (and socially displayed) choices made at the time of union formation have an impact on marital stability (“causal” effect of secularized choices) or whether they reflect general orientations that also affect marital stability (“selectivity” effect of secularized choices). The prevalence of the “causal” versus the “selectivity” effect of secularized choice has different implications for the future evolution of marital stability. Indeed, if selectivity prevails, the increasing diffusion of premarital cohabitation and civil marriage does not necessarily mean that divorce rates will rise in the near future.

We build our analysis on the multiprocess modelling approach developed by Lillard and colleagues (see, e.g., Lillard 1993; Lillard et al*.* 1995), and we model premarital choices as a set of simultaneous equations allowing for potentially correlated common unobserved factors. The decision to cohabit before marriage and to marry with a civil ceremony enter the equation of the hazard of divorce as explanatory factors which can be studied net of the effect of common factors giving rise to selectivity. We also model the effect of premarital cohabitation on the choice between civil marriage and religious ceremony. Our data—the survey called “Families and social subjects” (FSS) conducted in 2003 by ISTAT, the Italian National Statistical Office—provide suitable micro-level longitudinal information for this analysis.

The remainder of this article is structured as follows. In Sect. 2, we outline the development of civil marriage, cohabitation, and marital disruption in Italy, also taking into account a comparative perspective. In Sect. 3, we introduce our research questions and the hypotheses that we focus on. Data and methods are discussed in Sect. 4, while the results of our empirical analyses are presented in Sect. 5. Some concluding remarks are included in Sect. 6.

## Premarital Cohabitation, Civil Marriage, and Marital Disruption in Italy

In Italy, divorce is the final stage of a usually long process of separation and its subsequent legal recognition. While divorce was legalized in 1970, the minimum period of legal separation was reduced from 5 to 3 years only in 1987. The process leading to divorce after the decision to separate (or the de facto separation) may in fact last much longer. For these reasons, the proportion of marriages that fail is higher than suggested by the divorce rate alone. Marital disruption is better indicated by legal separation, as already suggested in previous studies (e.g., Castiglioni and Dalla Zuanna 2008).

In comparison with other western countries, the prevalence of divorce has been relatively low but clearly increasing. Considering all marriages, the number of separations rose from about 10,000 to about 80,000 in the decade 1995–2005 (Vignoli and Ferro 2009). For marriages celebrated in the early 1990s, the risk of divorce within the first 10 years has more than tripled with respect to marriages concluded in the early 1970s (Castiglioni and Dalla Zuanna 2008). At the cohort level, marital dissolution within 5 years of marriage increased from 3% for women born between 1953 and 1957 to 5% for the cohorts 1963–1967. Comparative figures for Western European countries are 10 and 15%, and for Northern European Countries they range from 15 to 33% (Liefbroer and Dourleijn 2006).

The incidence of premarital cohabitation has grown rapidly in recent decades. Based on the same data as those used for our subsequent analyses, we estimate that only about 1% of first marriages celebrated before 1974 were preceded by cohabitation. This percentage increased to 10 for marriages celebrated between 1984 and 1993, to 14 for those started in the following 5 years. It has reached 25% for more recent marital unions (years 1999–2003). Nevertheless, these features show that in Italy, direct marriage is still the most common way of starting the first union. In a comparative perspective, Italy continues to be characterized by traditional values with a strong propensity towards marriage. There is not a real “crisis” in marriage as an institution, and cohabitation remains a temporary experience that it is still not considered a real alternative to marriage (Rosina and Fraboni 2004). The persistent favorable attitude towards marriage is well highlighted by the fact that more than 80% of younger people do not consider it as an obsolete institution (see, for example GCD 2007). However, marriage is now increasingly postponed, and this phenomenon explains most of the reduction in crude marriage rates from 6 per thousand in 1990 to 4 in 2004.

Civil marriages have been spreading faster: during the early 1970s, the incidence of non-religious marriages (among all marriages) increased from 2 to 10%. In the following years, the increase continued steadily. Nowadays, more than one in three marriages is celebrated with a civil ceremony. The frequency is higher in the cities of the central and northern regions. However, the increase occurred more slowly than in other European countries for which data are available (Dittgen 1995). A possible explanation is that in Italy, a religious ceremony (i.e., almost exclusively, a Catholic ceremony) has the same effect as a civil ceremony with respect to the legal registration of marriage. It is not necessary to have a separate civil ceremony, as is the case, for instance, in France. Barbagli et al. (2003) suggest that changes in the kind of ceremony may be read in two different ways: as a sign of the lesser appeal of marriage as an institution or, on the contrary, as a new capacity of marriage to survive to the secularization process. These authors also specify that a relevant aspect of the growth of civil marriages is the diffusion of second marriages (5% of total marriages in 2000), which cannot be celebrated with a Catholic ceremony, and growing immigration (more than 50% of women born abroad chose civil marriage during the 1990s). In addition, unlike the other behaviors considered here, civil marriages do not necessarily prevail among younger individuals but their frequency increases with age. Rather than the effect of a possible period of cohabitation before marriage, this outcome is linked to the lower autonomy of younger people, more often bound to social and familial norms (Rosina and Fraboni 2004; Barbagli et al. 2003).

## Research Question and Hypotheses

In the international literature, with a strong emphasis on the United States, premarital cohabitation has attracted a great deal of attention as a potential cause of marital instability. The main starting point is that crude rates, or other descriptive statistics, suggest that divorce is more common among persons who cohabited before marriage, although the impact may vary markedly between countries depending on the prevalence of cohabitation within a society (Liefbroer and Dourleijn 2006). Also in the Italian case, the low diffusion of non-marital unions is associated with higher percentages of separations among couples who lived in premarital cohabitation (see Table 1).

**Table 1:** Percentage of first marriages ending with a legal separation according to premarital cohabitation and type of marriage ceremony, Italy

	Number of marriages	% Separated	% Divorced	% Civil marriage
Premarital cohabitation				
No	8,230 (91.7%)	8.0	4.3	11.0
Yes	746 (8.3%)	9.8	4.4	43.8
	8,976 (100%)	8.1	4.3	13.7
Marriage ceremony				
Civil	1,234 (13.7%)	12.6	6.2	
Religious	7,742 (86.3%)	7.4	4.0	
	8,976 (100%)	8.1	4.3	

Marriages celebrated after 1971

*Source* Own calculation on ISTAT FSS 2003

*Note* Weighted data (normalized post-stratification weights)

Higher rates of marital disruption among people who cohabited before marriage are usually explained in the literature by two main mechanisms: *selectivity* and *causation*.

The selectivity mechanism refers to the fact that premarital cohabitation is more frequently experienced by a selected group of people, who were already different in salient ways from the remainder of the population in terms of values and beliefs related to family life and marriage *before* the choice to cohabit. In the US context, some authors have underlined that individuals cohabiting before marriage are generally less oriented to perceive marriage as an “institution” compared to individuals who marry directly (Axinn and Thornton 1992; Thomson and Colella 1992) and that they are characterized by a stronger attachment to personal independence, a weaker commitment to marriage in general and fewer traditional attitudes and values that might act to stabilize a union (Bumpass et al. 1991; Carlson 1985; Sweet 1989). Moreover, Teachman and Polonko (1990) argue that cohabiting couples often marry because of pressure of family and peers. All these features make the decision to divorce more acceptable, leading to less stable marriages. Therefore, if the selectivity mechanism is at work, the higher divorce rates for premarital cohabitors would be explained through a spurious relationship: the effect of premarital cohabitation on divorce is apparent and might become weaker, or even disappear, when statistical controls for selectivity are introduced in the analysis (Lillard et al. 1995).

The second mechanism, *causation*, considers that individuals who cohabit before marriage might have developed (during cohabitation) different attitudes and value orientations that make success in marriage more difficult (Axinn and Thornton 1992). For example, cohabitation causes individuals to become more accepting of divorce because they develop a more individualistic perspective towards living as a couple and because they have evidence that reasonable alternatives to marriage exist (Thomson and Colella 1992). There might also be an effect running through secularized practices at the individual or couple level, as marriage itself boosts religious activities (Thornton et al. 1992; Stolzenberg et al. 1995). In other words, as pointed out for the Canadian case (Hall and Zhao 1995), the experience of cohabitation undermines the legitimacy of formal marriage, making divorce a suitable alternative when difficulties arise.

Previous analyses show that in Italy the influence of premarital cohabitation on union stability is negative, even in multivariate models (Liefbroer and Dourleijn 2006). However, causation may arise in the opposite direction: a period of cohabitation may be a first and useful screening mechanism (Teachman et al. 1991); it gives the chance to gain in advance information about the potential spouse and the kind of life the couple would have, therefore constituting a factor of protection against divorce (Lillard et al. 1995). Moreover, in the European context, non-marital unions with a poor chance of success will be terminated relatively quickly and will not be transformed into marriage (Liefbroer and Dourleijn 2006). As a consequence, those who survive until marriage will show lower risks of marriage dissolution. Following this perspective, Kulu and Boyle (2010) shows that premarital cohabitation, net of self-selection, decreases the risk of separation in Austria, supporting the notion of cohabitation as a “trial marriage” able to reinforce subsequent marital stability.

A third approach found in the literature, but that we will not consider in our analysis, is based on the idea that the increased risk of marital dissolution among persons who live with their future spouse before marriage may be explained by the longer time spent together. This hypothesis, starting from the assumption that marital dissolution increases with partnership duration, is not empirically supported and it has been repeatedly rejected in the literature, both in the US (Teachman et al. 1991; De Maris and Rao 1992) and in the UK (Berrington and Diamond 1999).

In parallel to what has been argued for premarital cohabitation, we can easily speculate that the value orientations at the root of the choice of civil marriage are not vastly different from those which make the choice of premarital cohabitation more likely. It is not by chance that after cohabitation, the probability of choosing a civil marriage increases substantially (see the last column in Table 1). The direct link between these two events has been underlined by Barbagli et al*.* (2003). As a consequence, the higher divorce rates among persons who experienced a civil marriage may be the outcome of the same selection process described previously. In this case, a spurious relationship may exist between the decision to have a civil marriage and premarital cohabitation, on the one hand, and the higher propensity to divorce, on the other. All these three choices would have been fostered by secularization. The alternative hypothesis considers the presence of a causal effect that may be positive (civil marriage increases divorce risk) or negative (civil marriage decreases divorce proneness). In the Italian context, where religious marriages are predominant, living in a civil marriage, i.e., in a minority group that could even be stigmatized, may be an experience that causes an ideational shift. On the one hand, it may push towards the radicalization of secularized and individualistic attitudes undermining marriage as an institution and, thereby, making divorce (or at least a legal separation) a more acceptable choice. On the other hand, it may strengthen the union, leading to a stronger consciousness of the importance of marriage and increased efforts to ensure marriage stability.

To sum up, we can reformulate the main substantive question of this article in new terms: how do secularized behaviors such as cohabitation and civil marriage affect subsequent marital stability? Which is the main mechanism, selectivity or causation? And, if the relationship is causal, is the sign of the causal relationship positive or negative?

## Data and Methods

Data for our analyses come from a multipurpose, nationally representative survey called “Famiglia e soggetti sociali” (“Families and social subjects”), FSS from now onwards. Carried out at the end of 2003 by ISTAT, the Italian National Statistical Institute, FSS contains retrospective information on life course trajectories, including data on the history of marital unions, cohabitations (followed by a marriage or not) and marital disruption, for a large sample of the resident population. FSS is the Italian component of the broader Generations and Gender Programme coordinated by the UNECE (Vikat et al. 2007).

We use a sub-sample of the main survey selecting 8,976 women born between 1940 and 1980 who were ever-married at the time of interview. The analysis was restricted to women because in the data we did not have relevant information about marriage, the type of marriage ceremony in particular, among separated and divorced men.

Moreover, we selected only first marriages celebrated after 1970, the year when the divorce law radically changed. In fact, legal separation was possible even before that year but it was extremely rare (Castiglioni and Dalla Zuanna 2008). The event of interest is the legal separation that marks the dissolution. Unfortunately, the date of separation was missing for 232 cases among those who declared the date of divorce. This means that 31% of dates were missing for separated women. We estimated the missing information according to the length of time between separation and divorce observed for non-missing cases clustered by level of education, marital status at the interview (remarried or not), and date of divorce (before or after 1987). A similar solution had already been adopted by Vignoli and Ferro (2009).

### Modelling Strategy

In order to investigate our main research questions, we used a multiprocess model composed of simultaneous equations allowing unobserved factors to be correlated across three decisions, i.e., whether to cohabit prior to marriage versus a direct marriage, whether to marry through a civil versus a religious ceremony, and whether and when to experience a legal separation. This kind of model, originally developed by Lillard (1993), is particularly useful for our purposes, since causal effects can be disentangled from selection effects as long as the functional form of the unobserved factors is assumed to be a multivariate normal distribution. Parameter estimates of the model can be obtained using aML, a software package for the estimation of advanced statistical models, which uses the maximum likelihood approach (Lillard and Panis 2003).

In detail, the model is composed of the following three equations (we remove the observation subscript *i*):

1 $$ \ln \mu \left( t \right) = \alpha_{0} (t) + \alpha_{1} Z_{1} + \alpha_{2} Z_{2} + \beta^{\prime}_{1} X_{1} (t) + \delta $$

2 $$ Pr\left( {Z_{1} = 1} \right) = \Upphi^{ - 1} \left( {\beta^{\prime}_{2} X_{2} + \varepsilon } \right) $$

3 $$ Pr\left( {Z_{2} = 1} \right) = \Upphi^{ - 1} \left( {\beta^{\prime}_{3} X_{3} + \alpha_{3} Z_{1} + \lambda } \right) $$

The first one is a hazard equation where the risk of legal separation at time *t* is a function of the baseline *α*_0_ (*t*), i.e., the pattern of duration dependence common to all individuals,^2^ an exogenous set of time-fixed and time-varying covariates (*X*_1_(*t*)), and two potentially endogenous decisions (premarital cohabitation *Z*_1_ and civil marriage *Z*_2_). For the first equation, episodes start (*t* = 0) at the time of marriage and end at the date of legal separation, if any. Otherwise, the episode is right-censored at the date of interview. Widowed respondents are censored at the date of the death of their spouse.

Equations 2 and 3 are binary regression models considering, respectively, the probability of cohabitation before marriage and a civil marriage as a function of a set of exogenous covariates *X*_2_ and *X*_3_. For these binary regressions we use a probit specification, i.e., Φ^−1^ is the inverse of the cumulative standard normal distribution.

To take selectivity into account, the three equations are jointly estimated through a model that allows for correlation in unobservables. We assume that the respective unobserved factors δ, ε, and λ all originate from the same tri-variate normal distribution. The three terms can be seen as representing, respectively, the woman’s propensity (constant over time) to separate (*δ*), to cohabit before marriage (*ε*), and to marry with a civil ceremony net of observed characteristics (*λ*). First, to avoid identification problems on the hazard scale, we impose the variance of δ as unitary (an alternative approach would require multiple marriages for a given individuals, a situation which is rarer in Italy and does not allow for the choice between civil and religious Catholic marriage unless the first marriage is civil). We then develop some robustness checks by letting the variance of *δ* vary.

As usual in probit equations, the variances of *ε* and *λ* are also fixed to unity. Therefore, we have:

$$ \left( {\begin{array}{*{20}c} \delta \\ \varepsilon \\ \lambda \\ \end{array} } \right) \sim N\left[ {\left( {\begin{array}{*{20}c} 0 \\ 0 \\ 0 \\ \end{array} } \right),\left( {\begin{array}{*{20}c} 1 & {} & {} \\ {\rho_{\delta \varepsilon } } & 1 & {} \\ {\rho_{\delta \lambda } } & {\rho_{\varepsilon \lambda } } & 1 \\ \end{array} } \right)} \right] $$

Generally speaking, a strong correlation between pairs of residuals means that some common unobserved factors (at individual level) simultaneously influence the two decisions. If all the correlations are statistically significant, *Z*_1_ and *Z*_2_ are endogenous and spurious effects arise, indicating that selectivity effects are present. Taking into account the three correlations, we can estimate the causal impact of premarital cohabitation and civil marriage on the risk of marital disruption net of selectivity.

### Background Variables

Exogenous variables and model specifications are presented in Table 2. First descriptive results are shown in Table 3 where we can see the increasing propensities among younger cohorts to cohabit and, in a lesser extent, to marry with a civil ceremony. The mean age of the sample at the interview is about 42 years.

**Table 2:** Variable definition and model specification

Variable	Categories	Equation		
		Separation (1)	Cohabitation (2)	Civil marriage (3)
Birth cohort	1940–1954 (ref.); 1955–1959; 1960–1964; 1965–1980	X	X	X
Educational attainment	Compulsory (lower secondary) or lower level (ref.); upper secondary level (high school); tertiary level (university degree)	X	X	X
Area of residence at the interview	Center-north Italy (ref.); south and islands	X	X	X
Couple’s age at union	Both woman and her husband lower than 25; both between 25 and 34 (ref.); man older (woman <25 and man ≥25 or woman <35 and man ≥35); woman older (man <25 and woman ≥25 or man <35 and woman ≥35); both 35 or higher	X		X
Current number of children (time-varying)	Childless (ref.); one child; two or more children	X		
Divorced parents	No (ref.); yes	X	X	X
Parents’ level of education	Low (both parents with primary school or lower level) (ref.); medium (at least one parent with lower secondary level); high (at least one parent with upper secondary level)	X	X	X
Number of brothers and sisters	0–1 siblings (ref.), 2 or more siblings		X	X
Husband’s level of education (at the beginning of the relationship)	Compulsory (lower secondary) or lower level (ref.); upper secondary level (high school); tertiary level (university degree)	X	X	X

**Table 3:** Percentage of women who cohabited before marriage, who preferred a civil marriage, and who separated according to various background characteristics

		% Among ever-married women	% Premarital cohabitation	% Civil marriage	% Legal separation	% Divorce
Birth cohort	1940–1954	25.4	4.4	11.9	8.2	5.7
	1955–1959	17.9	5.7	13.4	12.3	7.8
	1960–1964	19.6	7.3	15.4	8.4	4.0
	1965+	37.0	12.8	14.3	5.9	1.8
Educational attainment	Primary	13.1	6.4	11.6	3.7	2.4
	Lower secondary	44.1	7.7	13.8	7.9	4.0
	Upper secondary	32.0	8.7	13.6	9.6	4.9
	Tertiary	10.9	11.9	16.4	10.3	5.7
Area	Center-north Italy	64.3	9.5	15.2	9.7	5.4
	South Italy	35.7	6.2	11.2	5.2	2.2
Couple’s age at union	Both <25	26.4	12.4	14.2	10.2	5.8
	Both 25–34 years	30.4	6.8	12.6	7.6	2.8
	Man older	35.9	4.8	12.5	7.8	4.4
	Woman older	4.7	20.7	21.2	7.5	4.0
	Both 35+	2.5	8.8	26.3	9.2	4.8
Number of children at the interview	0	13.5	12.9	19.6	13.6	7.5
	1	28.4	9.3	14.4	11.2	6.1
	2+	58.1	6.8	12.1	5.3	2.6
Divorced parents	No	97.0	7.9	13.3	8.1	4.2
	Yes	3.0	20.4	28.9	10.0	5.9
Parents’ level of education	Low	64.3	6.4	11.7	6.3	3.3
	Medium	22.7	10.2	17.1	10.1	5.3
	High	13.1	14.5	17.9	13.6	7.7
Number of siblings	<2	43.1	8.3	13.6	8.8	4.6
	2+	56.9	8.4	13.9	7.6	4.0
Husband’s level of education	Primary	16.6	8.0	13.3	5.0	3.2
	Lower secondary	44.5	7.1	13.0	7.1	3.2
	Upper secondary	34.0	9.2	14.4	9.8	5.4
	Tertiary	4.9	14.4	17.5	16.4	10.5
Total		8,976	8.3	13.7	8.1	4.3

*Note* Weighted data (normalized post-stratification weights)

The effect of educational attainment on union stability is still not clear: it has been shown that educated women (tertiary level in particular) could show higher dissolution risks (Blossfeld et al. 1995; de Graaf and Kalmijn 2006; Hall and Zhao 1995), no effect at all (Bennet et al. 1988; Bracher et al. 1993; Lillard et al. 1995) or even lower risks (Berrington and Diamond 1999). The same uncertainty remains if we focus on Italy (De Rose 1992; Harkonen and Dronkers 2006; Liefbroer and Dourleijn 2006). On the other hand, we may imagine a positive effect of education on the propensity to cohabit and to start a marriage with a civil ceremony as the percentages in Table 3 suggest.^3^ To take the “couple perspective” into account, we also considered the husband’s level of education:^4^ Table 3 shows that highly educated husbands increase the incidence of secularized union formation and marital disruption.

With regard to the area of residence, socio-cultural and economic gaps between the north and the south of the country identify two potentially distinct patterns of secularized behaviors (Kertzer et al. 2009), as also suggested by crude rates in Table 3.

A union started very early in the life-course tends to be more fragile (Bennet et al. 1988; Berrington and Diamond 1999; Booth and Edwards 1985; Bracher et al. 1993; Murphy 1985). FSS data (Table 3) show that the lower the age at union (both for the woman and for her husband), the higher the incidence of separation and divorce but the lower the frequency of civil marriage.

Several studies reveal a reduced risk of marital dissolution among couples with children (Berrington and Diamond 1999; Coppola and Di Cesare 2008; Hoem and Hoem 1992; Murphy 1985; Weiss and Willis 1997; White 1990) even though there is also evidence that children have a destabilizing effect on unions (Boheim and Ermisch 2001; Chan and Halpin 2002; Hoem 1997). In our model, we include the current number of children as a time-varying variable.

Regarding the characteristics of the family of origin, we may expect a higher propensity to experience secularized union formation choices when the parents are highly educated (Bumpass and Sweet 1989; Di Giulio and Rosina 2007; Thornton et al. 1992) and when they are divorced (Thornton 1991). Even the size of family of origin could influence demographic behaviors through the amount of resources that parents can give to their children (Blake 1989), given that entry into cohabitation and civil marriage usually require lower resources than a Catholic marriage ceremony (Barbagli et al. 2003). The impact on marital stability of divorced parents has been confirmed several times (Amato and Keith 1991; Glenn and Kramer 1987; Hall and Zhao 1995) whereas parents’ education seems to be unimportant (Lillard et al. 1995).

## Results

The results of model estimation are shown in Table 4. Column (a) shows results considering independent (i.e., uncorrelated) equations, while column (b) shows the results of the joint multiprocess model. In Table 4, a coefficient above 0 implies a higher risk of disruption and a negative coefficient implies a lower risk of separation compared to the reference category.

**Table 4:** Estimates from independent and simultaneous equations model (standard errors in brackets) (women, Italy)

		Indep. Eq. a	Sim. Eq. b
Separation			
Baseline (slopes)	0–4 Years of marriage	0.09 (0.039)**	0.12 (0.039)***
	5–9 Years of marriage	0.13 (0.027)***	0.15 (0.028)***
	10 Years and more	−0.01 (0.010)	0.00 (0.010)
Constant		−6.19 (0.176)***	−6.78 (0.196)***
Birth cohort	1955–1959	0.59 (0.109)***	0.67 (0.119)***
	1960–1964	0.37 (0.124)***	0.44 (0.133)***
	1965+	0.78 (0.122)***	0.89 (0.133)***
Education	Upper secondary	0.11 (0.096)	0.11 (0.105)
	Tertiary	−0.09 (0.155)	−0.07 (0.166)
Area	South	−0.36 (0.093)***	−0.42 (0.099)***
Couple’s age at union	Both <25 years	0.43 (0.112)***	0.48 (0.122)***
	Man older	0.08 (0.103)	0.09 (0.111)
	Woman older	0.29 (0.200)	0.35 (0.221)
	Both 35+	0.45 (0.255)*	0.53 (0.287)*
Children	One child	−0.72 (0.095)***	−0.80 (0.102)***
	Two or more children	−1.49 (0.116)***	−1.64 (0.127)***
Divorced parents	Yes	−0.07 (0.207)	0.03 (0.235)
Parents’ level of education	Medium	0.44 (0.097)***	0.50 (0.105)***
	High	0.73 (0.122)***	0.83 (0.134)***
Husband’s level of education	Upper secondary	0.34 (0.093)***	0.40 (0.099)***
	Tertiary	1.02 (0.158)***	1.16 (0.174)***
Pre-marital cohabitation	Yes	0.30 (0.137)**	−0.11 (0.334)
Civil marriage	Yes	0.47 (0.100)***	0.30 (0.286)
Premarital cohabitation			
Constant		−1.79 (0.055)***	−2.52 (0.079)***
Birth cohort	1955–1959	0.12 (0.070)*	0.15 (0.097)
	1960–1964	0.23 (0.065)***	0.28 (0.097)***
	1965+	0.54 (0.056)***	0.76 (0.079)***
Education	Upper secondary	−0.10 (0.049)**	−0.14 (0.068)**
	Tertiary	−0.01 (0.074)	−0.01 (0.105)
Area	South	−0.23 (0.044)***	−0.30 (0.063)***
Divorced parents	Yes	0.40 (0.092)***	0.58 (0.128)***
Parents’ level of education	Medium	0.15 (0.049)***	0.21 (0.068)***
	High	0.33 (0.062)***	0.47 (0.088)***
Number of siblings	2 or more	0.12 (0.041)***	0.15 (0.057)***
Husband’s level of education	Upper secondary	0.06 (0.046)	0.08 (0.065)
	Tertiary	0.25 (0.089)***	0.36 (0.124)***
Civil marriage			
Constant		−1.31 (0.050)***	−1.76 (0.078)***
Birth cohort	1965+	−0.07 (0.038)*	0.03 (0.066)
Education	Upper secondary	−0.06 (0.044)	−0.11 (0.061)*
	Tertiary	−0.04 (0.067)	−0.05 (0.093)
Area	South	−0.13 (0.038)***	−0.22 (0.052)***
Couple’s age at union	Both <25 years	0.01 (0.050)	0.02 (0.067)
	Man older	0.04 (0.044)	0.06 (0.059)
	Woman older	0.17 (0.079)**	0.22 (0.107)**
	Both 35+	0.54 (0.097)***	0.71 (0.136)***
Divorced parents	Yes	0.42 (0.090)***	0.69 (0.116)***
Parents’ level of education	Medium	0.23 (0.044)***	0.35 (0.060)***
	High	0.18 (0.058)***	0.33 (0.081)***
Number of siblings	2 or more	0.06 (0.036)	0.10 (0.050)**
Husband’s level of education	Upper secondary	0.00 (0.041)	0.01 (0.058)
	Tertiary	0.03 (0.087)	0.11 (0.119)
Pre-marital cohabitation	Yes	1.03 (0.052)***	0.17 (0.338)
Residual correlation	(Separation–cohabitation) *ρ*_*δε*_		0.363 (0.246)
	(Separation–civil marriage) *ρ*_*δλ*_		0.25 (0.213)
	(Cohabitation–civil marriage) *ρ*_*ελ*_		0.90 (0.233)***
Log-likelihood (initial model with constant and baseline: −10766.23)		−10,083.50	−10,073.92
Number of cases		8,976	8,976

Significance: * >90%, ** >95%, *** >99%

Weighted data (normalized post-stratification weights)

Looking at the model with independent equations, we see a positive and significant relationship between experiencing civil marriage and premarital cohabitation and the risk of legal separation, as well as an association between the propensity to marry with a civil ceremony and the experience of premarital cohabitation. These findings show that multivariate models that account for some socio-demographic observed factors confirm the indications derived from descriptive analysis that premarital cohabitation and civil marriage weaken marriage stability. However, when we allow correlation between the heterogeneity components across the equations, none of the effects we mentioned turn out to be significant.^5^ This is the main result of our analysis: the increased risk of marital disruption for people who experienced premarital cohabitation can be entirely attributed to the selection of the most divorce-prone into cohabitation. Similarly, selectivity seems to be the explanation for the association between civil marriage and marital disruption as well, even though the coefficient of the effect of civil marriage on the hazard of disruption does not decrease in the simultaneous model. The results also suggest that union formation practices are endogenous in the separation equation, in the sense that they may depend on the partners’ commitment to marriage with direct effect on the stability of a marriage. Without controlling for the correlation among error terms, the impact of premarital cohabitation and civil marriage on divorce is therefore estimated in a biased way (Lillard et al. 1995).

In other words, among the three decisions considered in the analysis, there are no significant causal relationships, either *positive* (cohabitation and civil marriage do not increase the risk of disruption) or *negative* (cohabitation and civil marriage do not increase marital stability). We do not have evidence that during premarital cohabitation and civil marriage individuals tend to develop different attitudes and value orientations that make success in marriage more difficult.^6^

Considering the other covariates included in the models, we do not notice noteworthy changes when moving from independent to simultaneous equations. Looking at Table 4 we can briefly report the other results. Young cohorts (born after 1965) are more prone to cohabit before a marriage and to separate, but the cohort effect is not confirmed for the civil ceremony. The strong differences between the north and the south of Italy emerge clearly: living in the south of Italy means a lower propensity to divorce, to cohabit and to celebrate a civil marriage. In line with the previous literature, we find that earlier unions, i.e., those starting before 25 years of age, tend to be more fragile. On the other hand, the propensity to choose a civil marriage increases among unions starting later and, to a lesser extent, when the woman is older than her husband. The influence of a woman’s educational level becomes irrelevant or even disappears when we include the husband’s level of education, in the case of legal separation, or the parents’ level in the case of union formation. Generally speaking, our results indicate the importance of parental background and husband’s characteristics. In particular, the higher the educational level of the parents and husband, the stronger the propensity to experience the three behaviors (although our estimates cannot be interpreted as referring to the causal effect of education). Similarly, a positive association is found for women with divorced parents, although not in the case of marital disruption. A large number of siblings are associated with cohabitation but only slightly with civil marriage. Finally, as we expected, women with children are less likely to disrupt their marriages.

## Discussion

In Italy, several indicators suggest that religiosity is still widespread, and that the Roman Catholic Church still has a strong influence in the life of individuals. However, this apparent stability of religiosity is not sufficient to hinder the rapid increase in marital instability. In this article, we focused on two “external” manifestations of secularization in union formation practices: premarital cohabitation and civil marriage. We analyzed the effect of secularization in union formation practices on marital stability taking into account two mechanisms: selectivity and causation. Traditional (hazard) regression models show that cohabitation and civil marriage significantly increase the risk of marital disruption. Nevertheless, allowing correlation among unobserved factors able to influence the three decisions (legal separation, civil marriage, and premarital cohabitation), i.e., taking into account selectivity, the effect of premarital cohabitation is completely eliminated and the impact of civil marriage is not still statistically significant, suggesting that the apparent relationships that emerge in descriptive analysis and in independent models are spurious. In other words, selectivity appears to be the main explanation of the higher divorce rates among people who lived in premarital cohabitation or had a civil marriage.

Our results fail to support any causal relationship, either negative or positive. Net of selectivity, premarital cohabitation does not mean a useful screening period and no other positive effects emerge for people who married in a civil ceremony. At the same time, the experience of cohabitation itself and/or civil marriage is not some kind of black-box from which more individualistic and different attitudes emerge making success in marriage more difficult. The selection operates prior to union formation. Considering premarital cohabitation and civil marriage as indicators of a lower religiosity, we do not have evidence that “external” displays of religion conviction have a direct effect on marital stability. On the contrary, common unobserved causes are important. We can speculate that these causes are related to value orientations or unobserved individual propensities, similarly affecting “new” behaviors in the process of union formation, which underlie the higher divorce rates. We do not find evidence of an *event*-*based adaptation* of previously held value orientations towards a less family-oriented perspective. Following the Second Demographic Transition approach, this value orientation may be easily interpreted as a secularized attitude that lowers deference towards authorities, such as the Church, and raises individual autonomy. This latent perspective implies the “external” manifestation of secularization such as non-conventional modes of family formation and divorce.

Let us conclude with two observations. First, as we have already stressed in the introduction, the context plays an important role. Premarital cohabitation has different effects on the divorce risk in different societies and its impact on union stability depends on the prevalence of cohabitation within the specific country (Kiernan 2002). In countries where more rigid marriage norms prevail, cohabitation has a stronger effect on marital stability than in countries where marriage norms are weaker (Wagner and Weiss 2006). Liefbroer and Dourleijn (2006), ignoring selectivity, have shown that the relationship has a U-shape: it is stronger where cohabitation is not common, it is weaker in countries where the incidence is higher, but it becomes stronger again where marriage is the minority choice. The argument is that, where cohabitation is less common, cohabitors will probably constitute a highly selected part of the total population and the effect on marital stability is stronger. Italy, a country where cohabitation is still not as widespread as in other countries, is situated along the descending part of the U-shaped curve. Therefore, following the curve, we may predict that when cohabitation becomes more common in Italy, the differences in terms of divorce rates between persons who cohabited before marriage and those who did not should decrease substantially.

The second remark concerns the limits of this article. First, much of the identification of our model hinges upon the assumption that latent, unobserved factors are normally distributed. Identification would be improved in the case of multiple marital spells, and with the use of exclusion restrictions, i.e., different sets of variables for the three equations. Unfortunately, second marriages are very rare in our dataset and exclusion restrictions can only be partially applied because it is very difficult to justify that premarital cohabitation, civil marriage, and marital disruption depend on different factors. Reinhold (2010) attacks a similar problem with a non-parametric assumption on the distribution of unobserved factors. However, our problem is more complex than that of Reinhold because we tried to model three different decisions simultaneously, an approach that increases the difficulties of applying non-normal multivariate distribution. Aware that caution is needed when estimating hazard models based on single-spell data (i.e., only first marriages), Aassve et al. (2003) show through Monte Carlo simulations that allowing for a different fixed variance for the normal distribution of unobserved heterogeneity gives a robustness check. This was our robustness check (see note 6).

Second, we have only limited information on the characteristics of the spouse. Our results tend to confirm that separation is a decision taken by two individuals and the partner’s characteristics may strongly influence the stability of a marriage. However, we would need more detailed and accurate information to examine this point. We agree with the perspective of Bracher et al. (1993) that investigations of marriage stability would benefit from additional efforts to collect retrospective data on both spouses. Third, simultaneous models used in this analysis take into account unobserved factors that remain constant over the spells (marriage in our case), so we do not evaluate the causal effect net of time-varying unobserved heterogeneity. Fourth, as we maintain that the context is particularly important, territorial heterogeneity might have provided important insights (see, e.g., Lesthaeghe and Neidert 2006; Kertzer et al. 2009). Nevertheless, the current data and in particular the low number of dissolutions did not allow us to conduct a full analysis of territorial differences in the relationship between union formation practices and marital dissolution in Italy.

## Footnotes

1 According to the Roman Catholic Church, a civil marriage is not a marriage: “only those marriages are valid which are contracted in the presence of the local Ordinary or parish priest or of the priest or deacon delegated by either of them” (Code of Canon law n.1108 §1).

2 The baseline is a piecewise-linear spline with knots at the beginning of the fifth and the tenth year of marriage.

3 Given that more than 90% of the sample are more than 30 years of age, that the youngest woman is 23 years, and that it is very rare in Italy for a woman to continue her education after marriage, in our models we consider woman’s educational attainment (at the interview) as a time-constant covariate during the marriage episode.

4 In FSS data, the husband's level of education is considered only at the beginning of the engagement.

5 This result remains the same if we consider two sets of models estimated separately (here not shown), one for disruption and premarital cohabitation, the other for disruption and civil marriage.

6 We made several checks to test the robustness of our results. First, we included a variable considering the duration of premarital cohabitation as additional covariate. Second, we tried to estimate models excluding cases with missing information on legal separation. Third, we allowed for changes in the pre-fixed level of variance of the unobserved term in the hazard equation (from 0.6 to 1.4). In all cases, the sign, the magnitude, and the significance of the effects do not change substantially. Complete results are available upon request to the corresponding author.
